# Correlating the Interfacial Polar-Phase Structure to the Local Chemistry in Ferroelectric Polymer Nanocomposites by Combined Scanning Probe Microscopy

**DOI:** 10.1007/s40820-022-00978-3

**Published:** 2022-12-06

**Authors:** Jiajie Liang, Shaojie Wang, Zhen Luo, Jing Fu, Jun Hu, Jinliang He, Qi Li

**Affiliations:** State Key Laboratory of Power System, Department of Electrical Engineering, Tsinghua University, Beijing, 100084 People’s Republic of China

**Keywords:** Interfaces, Ferroelectric polymers, Nanocomposites, Scanning probe microscopy, Nano-infrared spectroscopy

## Abstract

Ferroelectric polymer nanocomposites possess exceptional electric properties with respect to the two otherwise uniform phases, which is commonly attributed to the critical role of the matrix–particle interfacial region. However, the structure–property correlation of the interface remains unestablished, and thus, the design of ferroelectric polymer nanocomposite has largely relied on the trial-and-error method. Here, a strategy that combines multi-mode scanning probe microscopy-based electrical characterization and nano-infrared spectroscopy is developed to unveil the local structure–property correlation of the interface in ferroelectric polymer nanocomposites. The results show that the type of surface modifiers decorated on the nanoparticles can significantly influence the local polar-phase content and the piezoelectric effect of the polymer matrix surrounding the nanoparticles. The strongly coupled polar-phase content and piezoelectric effect measured directly in the interfacial region as well as the computed bonding energy suggest that the property enhancement originates from the formation of hydrogen bond between the surface modifiers and the ferroelectric polymer. It is also directly detected that the local domain size of the ferroelectric polymer can impact the energy level and distribution of charge traps in the interfacial region and eventually influence the local dielectric strength.

**Supplementary Information:**

The online version contains supplementary material available at 10.1007/s40820-022-00978-3.

## Highlights

- A strategy that combines multi-mode scanning probe microscopy-based electrical characterization and nano-infrared spectroscopy is developed.
- A series of samples with different coupling agents and nanoparticles are characterized to unveil the local structure-property correlation of the interface in ferroelectric polymer nanocomposites.
- The formation of hydrogen bond between the surface modifiers and the ferroelectric polymer enhances the β-phase content and reduces the domain size of the interface, and hence promotes the local piezoelectric effect and breakdown strength.

**Supplementary Information:**

The online version contains supplementary material available at 10.1007/s40820-022-00978-3.

## Graphical abstract

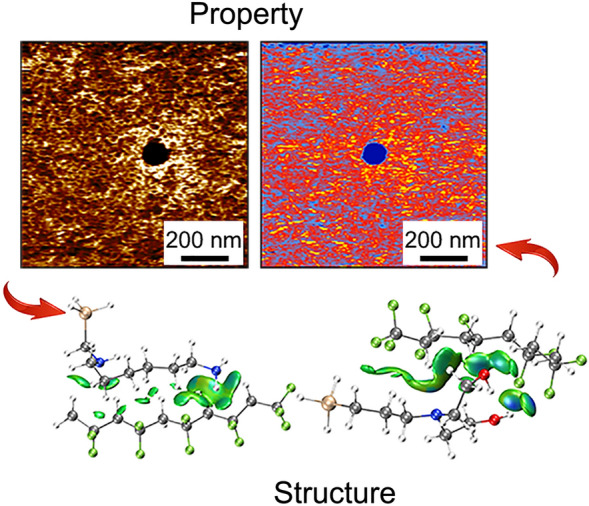

## Introduction

Ferroelectric materials are a class of functional dielectrics with spontaneous electric polarization that can be reversed with an applied electric field. As a subgroup of pyroelectric and piezoelectric materials, ferroelectrics also possess decent pyroelectric and piezoelectric responsive behaviors. By virtue of these intriguing properties, ferroelectric materials have been broadly exploited for various application areas such as energy storage capacitors, transducers, sensors, memories and electrocaloric refrigeration [1–4]. Up to date, the most explored two categories of ferroelectric materials are ferroelectric ceramics and ferroelectric polymers. Ferroelectric ceramics usually have strong spontaneous electric polarization, accompanied with relatively low voltage endurance, whereas ferroelectric polymers have high breakdown strength, low weight, good processability, but show weaker ferroelectricity compared with their inorganic counterpart [5–8]. Recently, a new category of ferroelectric materials named ferroelectric polymer nanocomposite have been developed, consisting of a ferroelectric polymer matrix embedded with nano-sized ferroelectric ceramic particles [9–16]. The ferroelectric polymer nanocomposite has aroused tremendous research interest not only because they combine the advantages of inorganic and polymeric ferroelectrics, but also due to the fact that they show unexpectedly greater performance than those predicted from the sums of the two otherwise uniform phases [15, 17]. Both theories [17–19] and experimental results [20–29] suggest that this phenomenon is attributable to the existence of the interfacial region, *i.e.*, a nanoscale transitional volume between the nanoparticles and polymer matrix [30]. However, the effect of matrix–particle interaction on the bulk material properties of the nanocomposite remains poorly understood. For instance, many previous studies attributed this effect to the interfacial interaction, or more specifically, the hydrogen bonds formed between the nanoparticles and polymer matrix [31–33], but there was no direct experimental evidence. Consequently, the design of ferroelectric polymer nanocomposite has largely relied on the trial-and-error method.

Most of the previous studies trying to understand the interface issue use bulk nanocomposite-based analyzing techniques to indirectly infer the interfacial property and structure [34–38]. For example, based on the result of Fourier-transform infrared spectroscopy (FTIR) and dielectric spectroscopy, it is proposed that the surface interaction of the embedded nanoparticles with the polymer matrix locally promotes the formation of *β*-phase configuration in the poly(vinylidene fluoride) (PVDF) and its copolymers, and thus significantly impacts the overall dielectric constant of the nanocomposite [17, 39–41]. This strategy is somewhat hypothetic, since it is usually challenging to completely distinguish the effect of interface from that of the intrinsic polymer/nanofiller properties under the framework of bulk material measurements, most of which are lack of sufficient spatial resolution.

Atomic force microscopy (AFM) [42], a kind of scanning probe microscopy with nanoscale spatial resolution, has become an important method for in situ testing of interface in polymer nanocomposites. In the past decade, researchers introduced AFM and its derivative working modes, such as electrostatic force microscopy (EFM), Kelvin probe force microscopy (KPFM), conductive-atomic force microscopy (C-AFM) and piezoelectric force microscopy (PFM), into the research field of ferroelectric polymer nanocomposites, with the research interest focusing on the electric polarization, dielectric response and space charge accumulation in the interfacial region [43–49]. But the structural origin to the exceptional electrical properties in the interface has been very rarely concerned in these studies. Later on, the mature of nano-infrared spectroscopy (Nano-IR) based on the AFM platform [50, 51] made it possible to test the local chemical structure of the interface. Very recently, the interfacial structure of a ferroelectric polymer nanocomposite was tested using Nano-IR [52]. Nonetheless, the probed structural information was not related to the electrical properties in the interfacial region. To date, in situ measurement of both chemical structure and electrical property in the same local region in ferroelectric polymer nanocomposites has not yet been reported, and the structure–property correlation of the interface remains largely unexplored.

**Fig. 1:**
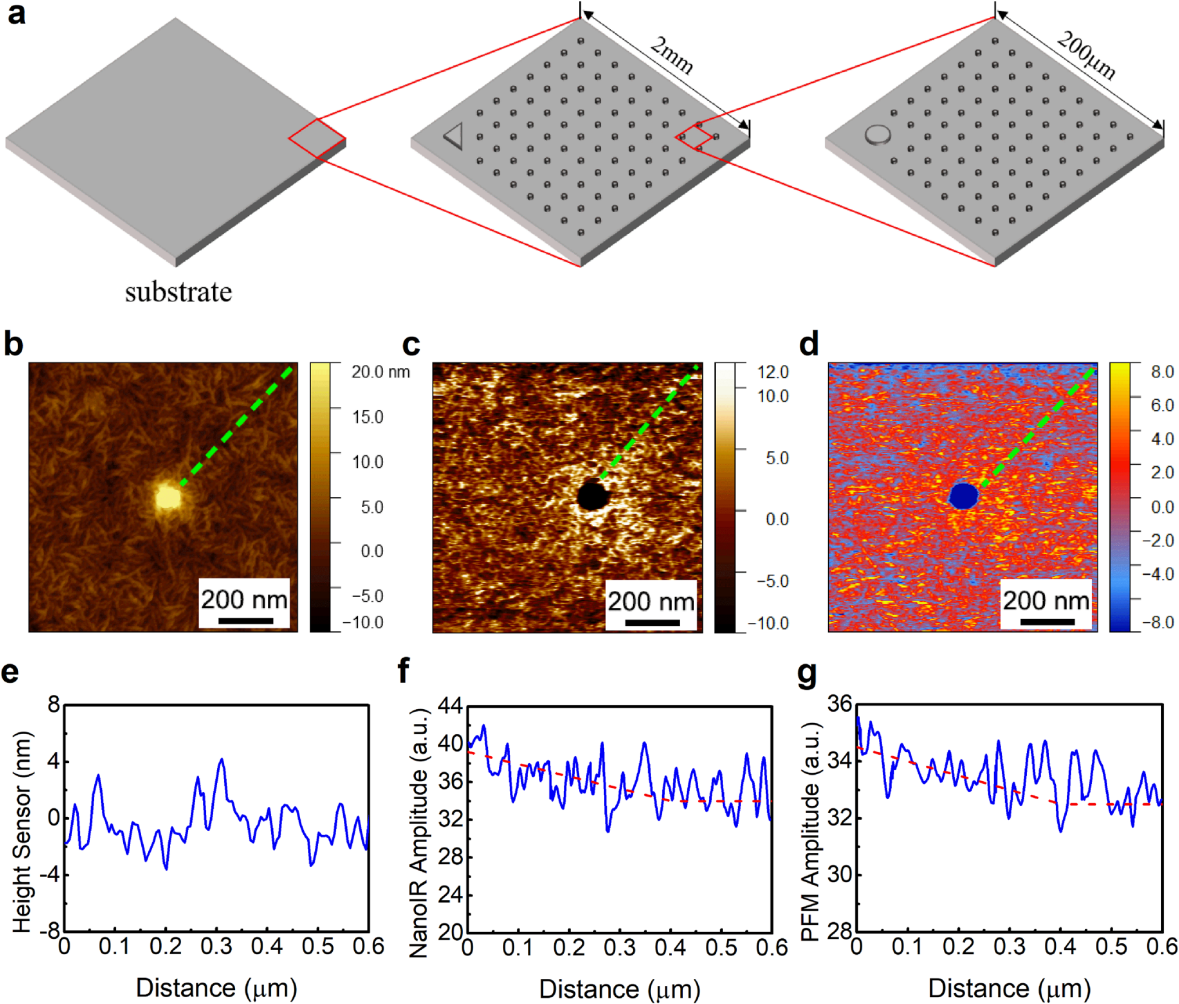
**a** Schematic of substrate structure. **b** The topography, **c** PFM mapping and **d** Nano-IR mapping at 1288 cm^−1^ of P(VDF-TrFE)/BTO nanocomposites. **e** Profile of b at the dashed line. **f** Profile of c at the dashed line. **g** Profile of d at the dashed line

In this study, we present a strategy to establish the micro-region structure–property correlation of the interface in ferroelectric polymer nanocomposites by combining multiple scanning probe microscopy-based high-resolution methods, *i.e.*, PFM, KPFM, C-AFM and Nano-IR. We demonstrate the effectiveness of this strategy by studying the change of local piezoelectric effect, charge trapping energy, breakdown strength and crystalline structure with varying the surface chemistry of the incorporated nanoparticles. The multi-mode scanning probe microscopy-based electrical characterization and Nano-IR are implemented by using two separate instruments, which are Bruker Dimension Icon system and Bruker Anasys nanoIR3 system. The Anasys nanoIR3 system does not support electrical property testing, and the topography scanning resolution of this system is lower than the Dimension Icon system due to the larger tip radius of curvature designed for Nano-IR test. Therefore, the topography and local electrical measurements are carried out on the Dimension Icon system. To make sure that the local measurements of electric property (PFM, KPFM and C-AFM) and crystalline structure (Nano-IR) would be performed on exactly the same area of the sample, we designed a gold-plated silicon substrate with a three-level protrusion matrix, as shown in Fig. 1a. With the optical microscope and topography microscope of these two instruments, we can locate the same area by referring to the marked protrusion.

## Experimental Section

### Sample Preparation

Barium titanate (BTO), magnesium oxide (MgO) and titanium oxide (TiO_2_) nanoparticles were purchased from Aladdin. Poly(vinylidene fluoride-trifluoroethylene) (P(VDF-TrFE)) powders were purchased from Sigma-Aldrich (70/30 mol%). The coupling agents trimethoxy(propyl)silane (TMPS), trimethoxy(decyl)silane (TMDS), trimethoxy(octadecyl)silane (TMOS), 1*H*,1*H*,2*H*,2*H*-Perfluorodecyltriethoxysilane (PFDTS), *N*'-(triethoxysilylmethyl)hexane-1,6-diamine (HMDATS) and 2-[2-hydroxyethyl(3-triethoxysilylpropyl)amino]ethanol (BHEAPTS) were all purchased from Aladdin. The P(VDF-TrFE) powders were dissolved in *N*,*N*-dimethylformamide (DMF) (Aladdin, GC, > 99.9%) and stirred over 6 h at the concentration of 20 mg mL^−1^. BTO nanoparticles mixed with the coupling agents were stirred in xylene (Aladdin, AR, 99.9%) under 100 ℃ for 12 h. Then, the mixtures were centrifuged under 1000 r/min to get modified nanoparticles. The nanoparticles were washed with toluene for 3 times and dried in vacuum for 8 h under 60 ℃. Nanoparticles were dispersed with DMF by a 30-min tip-type sonication (75 W) at the concentration of ~ 0.4 mg mL^−1^. The dispersion was spin-coated on a gold-plated substrate at 3000 r min^−1^ for 30 s immediately after the sonication. Then, the solution of P(VDF-TrFE) was spin-coated on the substrate at 2000 r min^−1^ for 30 s. The sample was annealed by heating under 70 ℃ for 6 h in a vacuum oven before the testing, in order to eliminate the influence of embedded nanoparticles on the modulus of the surrounding polymer. The typical thickness of the samples is 50 nm.

### Characterization

The Nano-IR measurements were performed on an Anasys nanoIR3 (Bruker) system with a quantum cascade laser (QCL) source (800–1900 cm^−1^) or a firefly laser source (2700–3600 cm^−1^) in tapping AFM-IR mode. Other AFM-based measurements were conducted on a Dimension Icon (Bruker) system. The topography was measured by SCANASYST-AIR probes in the ScanAsyst mode. The piezoelectric amplitude was measured by SCM-PIT-V2 probes in the contact resonance PFM (CR-PFM) mode with an electrical excitation of 5–10 V at the contact resonance frequency. The elastic modulus was measured by RTESPA-300 probes in the Peakforce Quantitative Nanomechanical Property Mapping (QNM) mode. The nano-isothermal surface potential decay measurement (Nano-ISPD) was measured by SCM-PIT-V2 probes in the KPFM mode. A line passing through the spot to be measured was scanned repeatedly. The tip bias was first set to 5 V until the electric potential of the line stabilized and then set to 0 V. The change of the electric potential was recorded. The breakdown behavior was measured by SCM-PIT-V2 probes in the C-AFM mode. The tip bias was applied by a nanoscope V controller-Signal Access Module (SAM V) to raise the limit of the voltage, with an increasing velocity of 1 V s^−1^. Thermal gravity analysis (TGA) was performed with a TA Q500 instrument, and the typical sample amount for TGA was 5 mg. The samples were tested from 30 to 600 ℃ with a heating rate of 10 ℃ min^−1^. The solid ^19^F nuclear magnetic resonance (NMR) was obtained by a JEOL-ECZ600R instrument.

### Calculation

The grafting rates were figured out by Eq. (1):

1 $$C = \frac{{6m_{{{\text{loss}}}} \rho _{{{\text{BTO}}}} N_{A} }}{{(m_{0} - m_{{{\text{loss}}}} )M_{{{\text{modifer}}}} d_{{{\text{BTO}}}} }}$$

where *C* is the grafting rate, *m*_loss_ is the lost mass of sample during the TGA progress, *m*_0_ is the mass of sample before TGA, *ρ*_BTO_ is the density of BTO, *d*_BTO_ is the average diameter of BTO nanocomposites, *M*_modifier_ is the relative molecular mass of the modifier, and *N*_A_ is the Avogadro constant.

The binding energy (*E*_b_) between modifiers and VDF part were calculated by independent gradient model (IGM) isosurface analysis with Multiwfn 3.6. *E*_b_ was figured out by Eq. (2):

2 $$E_{b} = E_{{{\text{complex}}}} - (E_{{{\text{modifier}}}} + E_{{{\text{VDF}}}} )$$

where *E*_complex_, *E*_modifier_ and *E*_VDF_ stand for the total energies of complexes, modifier and VDF, respectively.

## Results and Discussion

### Interfacial *β*-phase Content and Piezoelectric Effect

#### PFM and Nano-IR Measurements

We first applied vertical PFM measurement to the surrounding area of nanoparticles in a ferroelectric polymer nanocomposite sample. The polymer employed in this study is P(VDF-TrFE), *i.e.*, a PVDF-based random copolymer with trifluoroethylene as the chaining defect to stabilize the polar phase (all-*trans* conformation). And the fillers are BTO nanoparticles (100 nm diameter in average), a typical perovskite-type ferroelectric compound. The nanocomposite sample was prepared on the gold-plated silicon substrate by a two-step spin-coating process [46]. P(VDF-TrFE) powders and BTO nanoparticles were dissolved/dispersed in DMF before spin-coating. We successively spin-coated the BTO suspension and the P(VDF-TrFE) solution on the substrate. The thickness of the PVDF layer was controlled at approximately 50 nm by adjusting the concentration of the solution to make the nanoparticles partially exposed (Fig. 1b). According to the principle of PFM, the gauged amplitude is proportional to the piezoelectric coefficient in vertical direction (*d*_33_) of the sample, so we can use the PFM amplitude to estimate the piezoelectric coefficient of the sample. The mapping of PFM signal is shown in Fig. 1c. It is clear that the signal intensity of the area surrounding the nanoparticle is higher than that of the polymer matrix far away from the nanoparticle. A dashed line is marked in the mapping of PFM signal for the profile analysis. The PFM signal intensity along the dashed line tends to first decrease with distance away from the surface of the nanoparticle and then reaches a constant value at about 400 nm (Fig. 1f). In addition, this trend has no evident correlation with the local topography of the sample (Fig. 1e) or the local elastic modulus surrounding the nanoparticles (Fig. S1). These results suggest that the incorporation of the nanoparticle enhances the piezoelectricity of the surrounding polymer matrix within a range about 400 nm.

To understand the enhancement of PFM signal in the vicinity of the nanoparticles, we performed Nano-IR measurement on the same area of the sample. Nano-IR technique combines AFM platform with the photo-thermal induced resonance. When the sample is irradiated by an IR laser with a certain wavenumber, the corresponding chemical bonds or functional groups in the sample will absorb the IR energy and vibrate. Such vibration will cause a thermal expansion of the sample, and the expansion will be detected by the cantilever of the probe. Our experiment was implemented by using a QCL source. Considering that the piezoelectric effect of P(VDF-TrFE) mainly stems from the *β*-phase (all-*trans* conformation), we used an IR laser at 1288 cm^−1^, which corresponds to the signature of *β*-phase in P(VDF-TrFE) [52], to irradiate the sample. The mapping of IR signal is shown in Fig. 1d, where the intensity of *β*-phase is increased in the matrix–particle interfacial region. Interestingly, the profile analysis of IR amplitude (Fig. 1g) shows good agreement with that of the PFM amplitude, implying that the enhancement of PFM signal in the neighborhood of the nanoparticles is related to the increasing *β*-phase content in the surrounding polymer.

#### Correlation between the Local Structure and Property

**Fig. 2:**
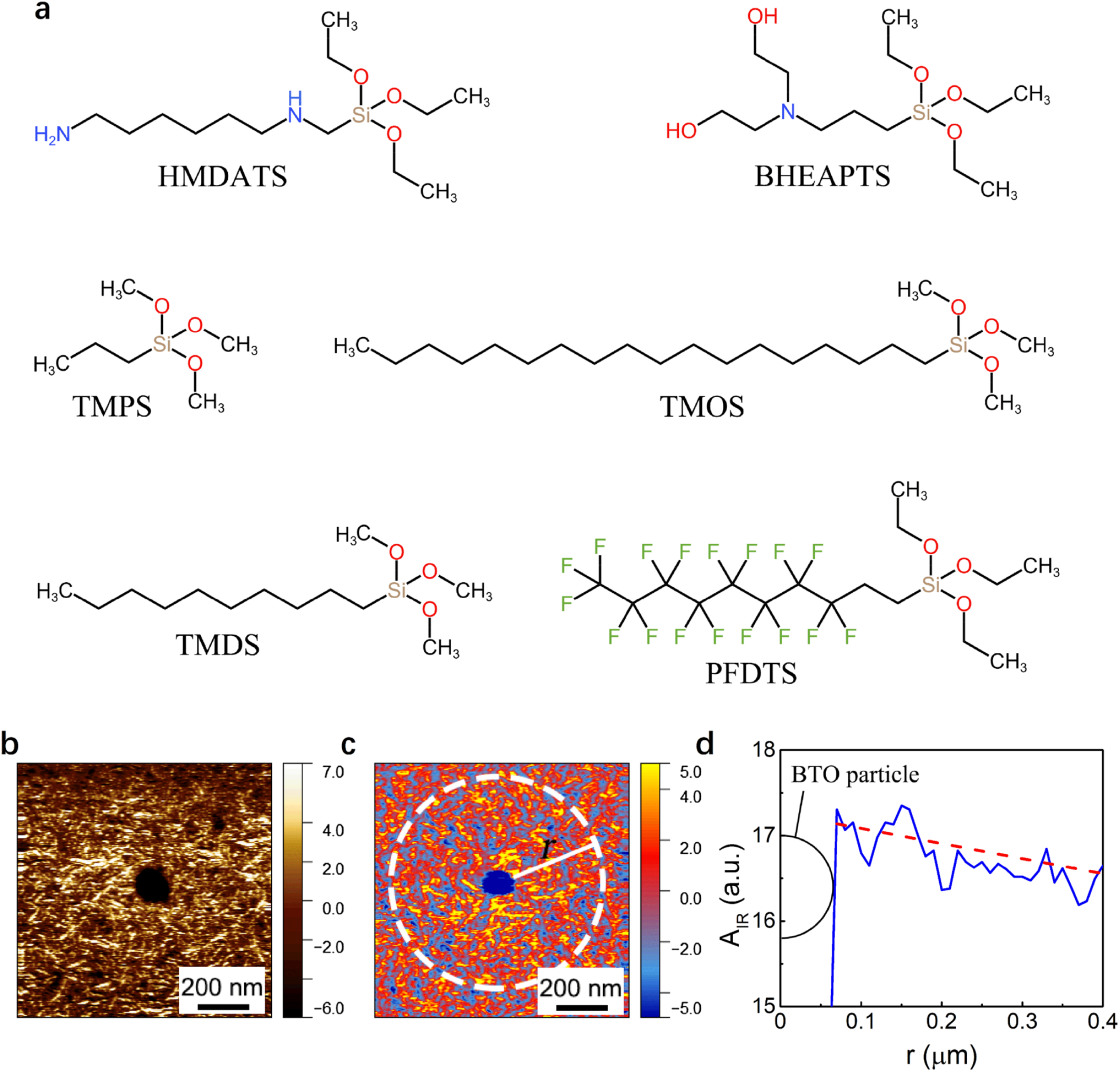
**a** The chemical structures of silane coupling agents. **b** The PFM mapping and **c** Nano-IR mapping at 1288 cm.^−1^ of P(VDF-TrFE)/BTO@TMDS nanocomposites. **d** *A*_IR_ of **c** (blue line) and its linear fitting (red dashed line). (Color figure online)

**Fig. 3:**
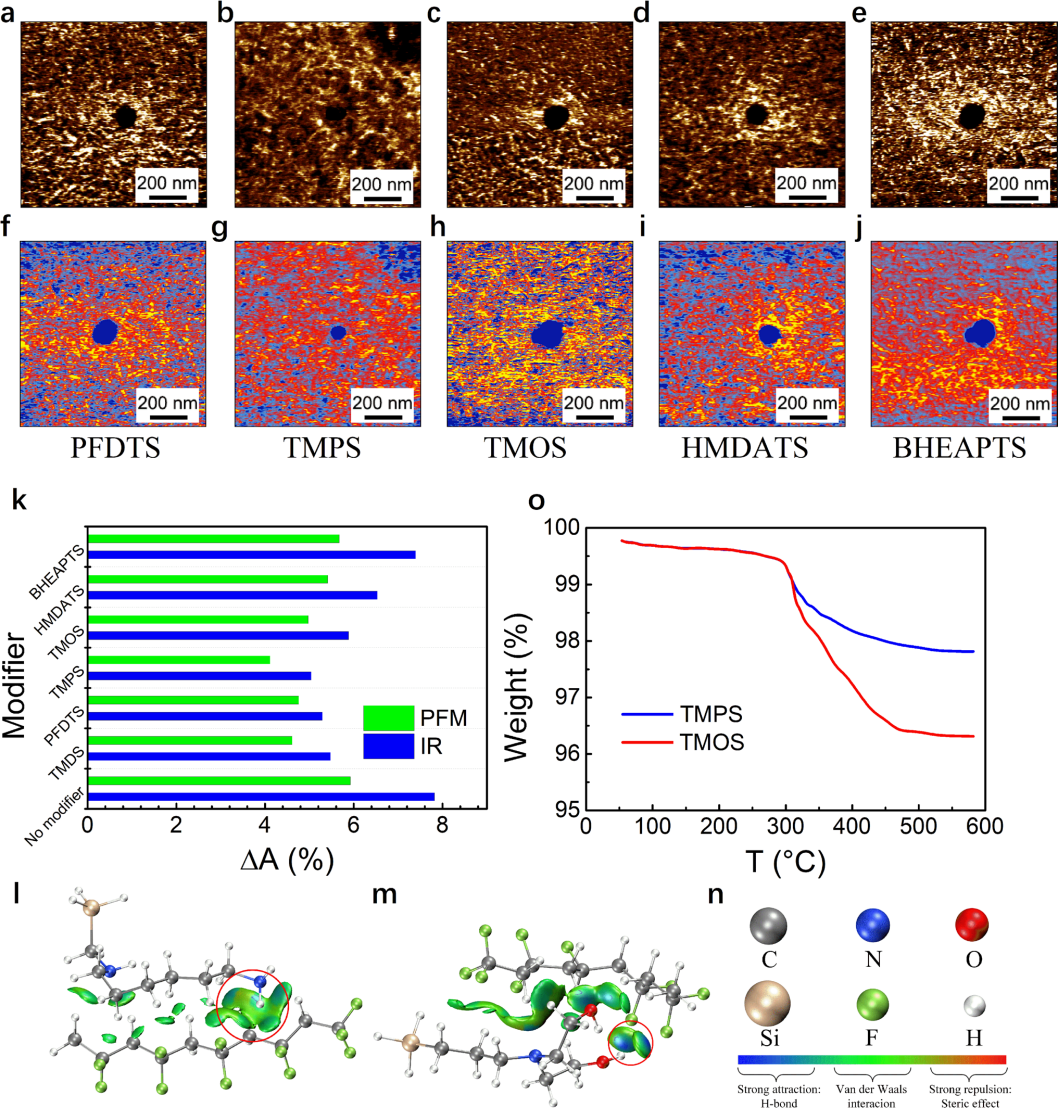
**a**–**e** The PFM mappings and **f**–**j** Nano-IR mappings at 1288 cm^−1^ of samples with BTO particles modified by different modifiers. **k** Δ*A*_PFM_ and Δ*A*_IR_ of samples with BTO particles modified by different modifiers. Schematics of IGM results of **l** HMDATS and **m** BHEAPTS with VDF. **n** Legend of l and m. **o** TGA results of BTO particles modified by TMPS and TMOS

To establish the correlation between the crystalline structure and piezoelectric effect of the interfacial region, we next investigated the impact of changing the surface chemistry of the nanoparticles on the local PFM and Nano-IR intensity obtained surrounding the nanoparticles. It is reported that the surface ligands of incorporated nanoparticles can influence the chain conformation and crystalline behavior of the polymer matrix [31, 53, 54], although the underlying correlation remains largely unknown. In this study, a series of coupling agents with different chain length and end group were utilized to surface-functionalize the BTO nanoparticles, *i.e.*, TMPS, TMDS, TMOS, PFDTS, HMDATS and BHEAPTS. The chemical structures of the coupling agents are shown in Fig. 2a. The nanocomposites containing the BTO nanoparticles modified with these coupling agents are termed as P(VDF-TrFE)/BTO@C-A, where C-A stands for the coupling agent. PFM and Nano-IR measurements were carried out on all the nanocomposite samples filled with BTO nanoparticles modified by the above-mentioned six types of coupling agents (Figs. 2b–d and 3**)**. To minimize the error of data and to make them comparable between different nanocomposite samples, the signal intensity was averaged and quantitatively defined in a way described as follows. We name the distance between a certain point and the center of the particle as *r*, and then, all the points with the same *r* form a circle (Fig. 2c). The average values of PFM amplitude and IR amplitude of all the points on a circle are termed as *A*_PFM_ and *A*_IR_, respectively. We plot *A*_PFM_ versus *r* and *A*_IR_ versus *r* where *A*_PFM_ and *A*_IR_ decrease with increasing *r*, and these curves fluctuate less than the curves of profile analysis. A curve of *A*_*IR*_ versus *r* is shown in Fig. 2d as an example. We then linearly fit these curves into straight lines. By comparing the averaged amplitude value of the first point (*A*_0_) on the straight line starting from the boundary of the nanoparticle and that 400 nm away from the first point (*A*_400_), we can figure out the increment ratio by using the equation [(*A*_0_ − *A*_400_) × 100%] / *A*_400_. The increment ratios for the PFM and Nano-IR signals are named as Δ*A*_PFM_ and Δ*A*_IR_, respectively. For instance, in Fig. 2d, the Δ*A*_IR_ is 5.47%.

The Δ*A*_PFM_ and Δ*A*_IR_ of all the nanocomposite samples, including the six nanocomposites filled with surface-functionalized BTO nanoparticles and a control nanocomposite filled with bare BTO nanoparticles, are extracted from the corresponding mappings (Fig. 3a–j) and are summarized in Fig. 3k. It is evident that the variation of Δ*A*_PFM_ in all the samples follows exactly the same pattern to that of the Δ*A*_IR_, again confirming that the enhancement of PFM signal in the interfacial region is related to the increasing *β*-phase content in the local area. To unveil the underlying structure–property correlation, we further analyzed the data by groups. Note that previous studies proposed that the molecular chain length and functional groups of the surface modifier as well as their interaction with the polymer chains (*e.g.*, hydrogen bonding) might possibly influence the interfacial property in the nanocomposites [31, 55, 56], although there has been no direct experimental evidence. We first compared the three samples containing TMPS-, TMDS- and TMOS-modified BTO nanoparticles. These three coupling agents have very similar structures, and the only difference is the molecular chain length. The result shows that the nanoparticle decorated with the modifier possessing a longer molecular chain has a greater impact on both the *β*-phase content and piezoelectric effect, leading to larger Δ*A*_PFM_ and Δ*A*_IR_. In the second comparison, we analyzed the nanocomposites containing TMDS- and HMDATS-modified BTO nanoparticles. The molecular chain of TMDS is longer than HMDATS who ends with the amino group, but the Δ*A*_PFM_ and Δ*A*_IR_ of the former nanocomposite are even lower, which seems contradictory to the result of the first group of comparison, suggesting that the molecular chain length of the surface modifier is not a decisive factor determining the local crystalline structure and piezoelectric effect. To examine if the polarity of chemical bonds or functional groups on the modifiers plays a critical role, we then carried out a third comparison between the nanocomposites containing TMDS- and PFDTS-modified BTO, where the C-F bonds in PFDTS have stronger polarity than C-H bonds in TMDS. It turns out that the two nanocomposites exhibit similar Δ*A*_PFM_ and Δ*A*_IR_, which means the influence on the interfacial structure and property is not simply related to the functional group polarity. We noticed that the three nanocomposites with the highest Δ*A*_PFM_ and Δ*A*_IR_ are those containing bare BTO, HMDATS-modified BTO and BHEAPTS-modified BTO. A common feature of these three nanocomposites is that the amino groups in HMDATS and hydroxyl groups in BHEAPTS or on the surface of bare BTO can all form hydrogen bonds with the fluorine atoms in P(VDF-TrFE) [57, 58].

#### Verification of Hydrogen Bonding

To confirm the existence of hydrogen bonds, we first performed ^19^F NMR on the two samples with and without BTO nanoparticles (Fig. S2). The signal peak representing F in CF_2_ groups shifts downfield, demonstrating a higher electron cloud density, which proves the existence of hydrogen bonds in the nanocomposites [59]. Furthermore, to make sure that the hydrogen bonds are indeed located in the interfacial region, we measured the Nano-IR spectra of different samples between 3200 and 3600 cm^−1^ in the interface and the matrix (Fig. S3), where the signal peak at ~ 3393 cm^−1^ corresponds to the stretching vibration of hydroxyl groups. With the decoration of BHEAPTS, the peak center shifts to a lower wavenumber compared with that functionalized with TMPS, indicating the formation of more hydrogen bonds [56, 60]. By contrast, the polymer matrix has no obvious signal in this range of wavenumber. These results serve as strong evidence that the hydrogen bonds form in the local interfacial region in a hydroxyl group modified nanoparticle composite. It is thus reasonable to believe that the formation of hydrogen bond is the critical factor influencing the *β*-phase content and piezoelectricity of ferroelectric polymer nanocomposites. This is supported by the computational simulation via the IGM method. The binding energy of HMDATS and BHEAPTS with the VDF part of P(VDF-TrFE) was calculated (Fig. 3l, m). According to the calculation, the binding energy of HMDATS with the VDF part of P(VDF-TrFE) is -94.91 kJ mol, whereas the binding energy of BHEAPTS with the VDF part is -143.60 kJ·mol, indicating that BHEAPTS can form a stronger interaction with the polymer chains. The simulation is in accordance with the Δ*A*_PFM_ and Δ*A*_IR_ results.

The impact of hydrogen bond formation in P(VDF-TrFE)/bare BTO nanocomposite on the interfacial structure and property can also explain the result of nanoparticles modified by TMPS, TMDS and TMOS, as described in the above-referenced first group comparison. The quantity of hydroxyl groups on the surface of bare BTO nanoparticles would decrease upon the surface modification due to the fact that the coupling reaction consumes the hydroxyl groups, as confirmed by the result of TGA (Fig. 3o). Combining the TGA results and the relative molecular weight of TMPS and TMOS, the grafting ratio of TMPS on the BTO nanoparticles was calculated to be 39.8% higher than that of TMOS, which means the remaining hydroxyls on BTO nanoparticles modified by TMPS are less. The difference in grafting ratio is probably resulted from the different molecular chain length of the modifier, which poses steric hindrance against the coupling reaction. Therefore, the Δ*A*_PFM_ and Δ*A*_IR_ results of the nanocomposites involving TMOS are higher than that involving TMPS. According to the profile analysis, the modulus does not change obviously with the distance, implying that the variation of PFM signal is irrelevant to the elastic modulus of the material.

**Fig. 4:**
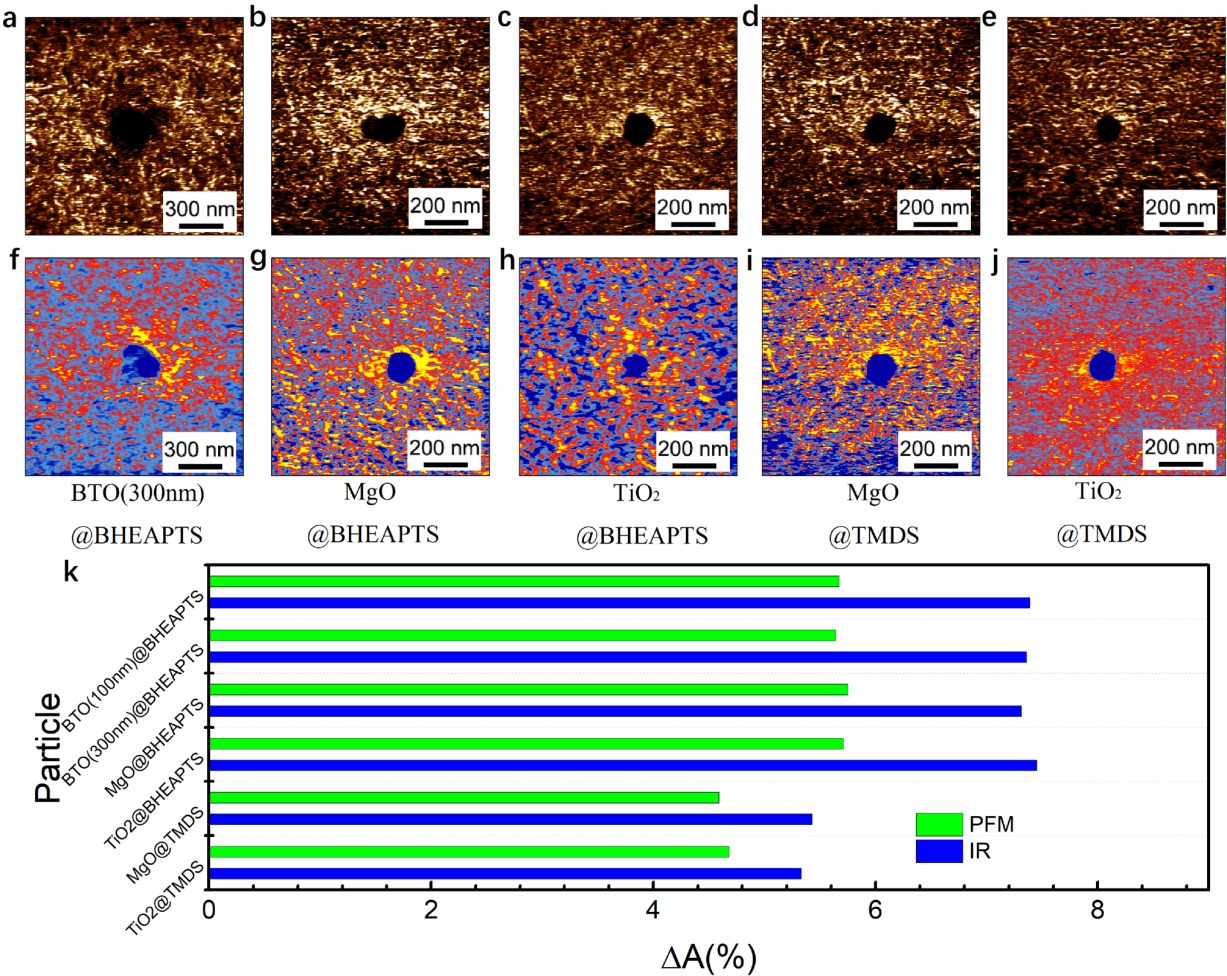
**a**–**e** PFM mappings and **f**–**j** Nano-IR mappings at 1288 cm^−1^ of samples with different particles modified by BHEAPTS and TMDS. **k** Δ*A*_PFM_ and Δ*A*_IR_ of samples with different particles modified by different modifiers

It is also interesting to know if the type of nanoparticles and the particle size influence the local structure and property in the interfacial region. We performed PFM and Nano-IR measurements on some other nanocomposite samples based on P(VDF-TrFE) incorporated with different types and sizes of nanoparticles, including MgO, TiO_2_ and BTO (300 nm diameter in average). The results are shown in Fig. 4a–j. There is no apparent difference between the nanocomposites filled with different nanoparticles with the same modifier. With the above results in mind, it is safe to draw a conclusion that the intensity of hydrogen bond plays the critical role in determining the *β*-phase content and piezoelectric effect of ferroelectric polymer nanocomposites.

### Interfacial *β*-phase Domain Size and Breakdown Strength

**Fig. 5:**
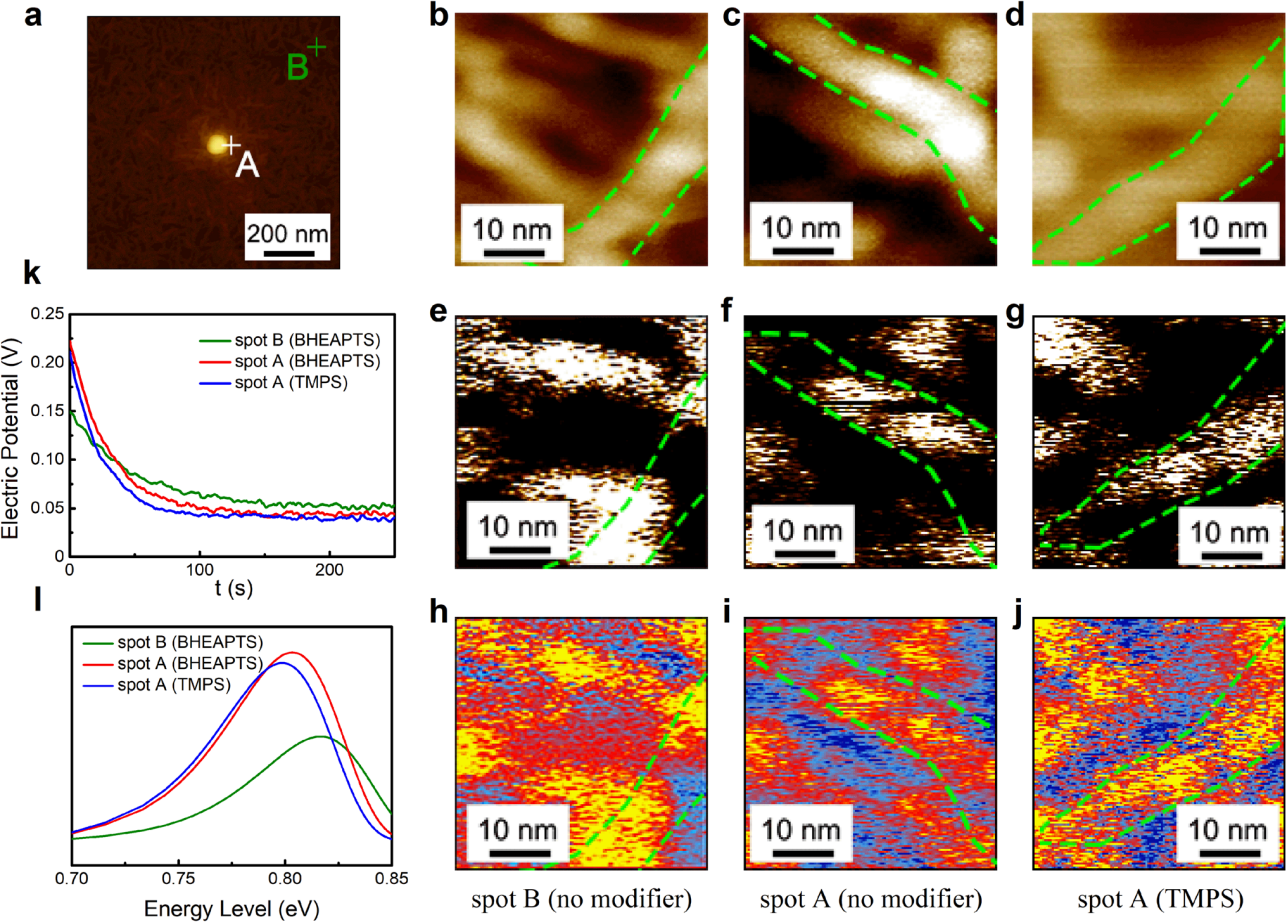
**a** Schematic showing the location of spot A and spot B. **b**–**d** Topography, **e**–**g** PFM mappings and **h**–**j** Nano-IR mappings at spot A and spot B in different samples. **k** Nano-ISPD results at spot A and spot B in P(VDF-TrFE)/BTO@BHEAPTS. **l** Local charge trap-level distribution obtained from **k**

In addition to the *β*-phase content, the *β*-phase domain size is another structural parameter that has major impact on the electric properties of ferroelectric polymers. To learn the correlation between the interfacial interaction and the domain size in the ferroelectric polymer nanocomposites, we performed PFM and Nano-IR measurements in a small area (50 × 50 nm^2^), near (spot A in Fig. 5a) and far away from the particle (spot B) on two different nanocomposites, *i.e.*, P(VDF-TrFE)/bare BTO (more hydrogen bonds) and P(VDF-TrFE)/BTO@TMPS (less hydrogen bonds). The results are shown in Fig. 5b–j. The worm-like crystals in the polycrystalline structure of the P(VDF-TrFE) are clearly presented in the mapping of topography (Fig. 5b–d). The corresponding PFM mappings reveal the domain size of the polymer given the fact that the domain boundary has weaker piezoelectric response than the domain region, *i.e.*, the continuous bright region most likely stands for an individual domain, and the dark region in between is the domain boundary. The Nano-IR images are in agreement with the PFM mappings, where the domain boundary shows weaker signal of *β*-phase due to the less ordered chain orientation in the boundary region. Apparently, the domain sizes at spot A of both the two nanocomposite samples are smaller than that at spot B. Besides, the domain size at spot A in the TMPS-decorated sample is larger than that in the bare one. Note that the nanoparticle modified by TMPS can form less hydrogen bonds with the polymer matrix than the bare BTO, causing a weaker interfacial interaction. These results corroborate that the interfacial interaction contributed by hydrogen bonding can lead to reduced domain size in the vicinity of the interfacial region [32, 61, 62].

To explore the influence of domain size on the charge transport in the interfacial region, we further performed Nano-ISPD developed in our previous study [49] at spot A and spot B in a nanocomposite sample (Fig. 5k), *i.e.*, P(VDF-TrFE)/BTO@BHEAPTS. The Nano-ISPD is on the basis of the KPFM method (see Supporting Information for details). We calculated the local trap level distribution and trap density (Fig. 5l) according to the model of ISPD [63] to quantitatively compare these results. The significantly higher peak of the curve at spot A clearly suggests that more traps are introduced in the interfacial region, *i.e.*, the trap density at spot A (interfacial region) in the BHEAPTS-decorated sample is 80.2% higher than that at B (polymer matrix). This is ascribed to the increased domain wall proportion containing abundant trapping sites for charge carriers, which also leads to overall slightly lower trap energy level of the interfacial region (~ 0.13 eV) in comparison with that of the matrix. Finally, to confirm the effect of changing trap state on the dielectric breakdown strength of the interfacial region, we performed C-AFM to measure the micro-region current as a function of the voltage bias at spot A and spot B in the BHEAPTS-decorated sample (Fig. S4, Supporting Information). The voltage bias at which the abrupt increase in current is observed is considered the breakdown point. Assuming that the thickness of the film is uniform, the breakdown strength at spot A enhances by approximately 15% than that at spot B. This result implies that the decreased domain size enhances the dielectric breakdown strength. The increased trap density causes more charges to get trapped, and the increased domain wall proportion results in a longer breakdown path. Both the two facts inhibit the occurrence of dielectric breakdown.

## Conclusions

In conclusion, we have demonstrated a strategy combining PFM, KPFM, C-AFM and Nano-IR to correlate the local electric property of interfacial region to the local crystalline structure in ferroelectric polymer nanocomposites. The results showed that the type of surface organic modifiers on the nanoparticles can significantly influence the *β*-phase content, the piezoelectric effect and the charge transport behavior of the polymer matrix surrounding the nanoparticles. The strongly coupled local *β*-phase content, piezoelectric effect and computed bonding energy suggested that the piezoelectricity enhancement could be attributed to the formation of hydrogen bond between the surface modifiers and the ferroelectric polymer. The domain size of the ferroelectric polymer near the matrix–particle interface was found to be smaller than the polymer matrix, which leads to significantly increased charge trap density and slightly lower trap energy level in the interfacial region, and hence improved local dielectric strength. These results imply that the strategy reported in this work is effective in understanding the structure–property correlation of the interfacial region, which is anticipated to better guide the design and fabrication of advanced ferroelectric polymer nanocomposites.

### Supplementary Information

Below is the link to the electronic supplementary material.

Supplementary file1 (PDF 176 KB)
