# Supplementary material for: Correlating the Interfacial Polar-Phase Structure to the Local Chemistry in Ferroelectric Polymer Nanocomposites by Combined Scanning Probe Microscopy
Source: Nanomicro Lett. 2022 Dec 6;15:5. doi: 10.1007/s40820-022-00978-3 (PMC9727024; doi:10.1007/s40820-022-00978-3)
Supplement: Supplementary file 1 — Supplementary file1 (PDF 176 KB) [file 40820_2022_978_MOESM1_ESM.pdf]

Supporting Information for

Correlating the Interfacial Polar-Phase Structure to the Local Chemistry in Ferroelectric Polymer Nanocomposites by Combined Scanning Probe Microscopy

Jiajie Liang[1], Shaojie Wang[1], Zhen Luo[1], Jing Fu[1], Jun Hu[1], Jinliang He[1], and Qi Li[1, *]

[1]State Key Laboratory of Power System, Department of Electrical Engineering, Tsinghua University, Beijing, 100084, P. R. China

*Corresponding author. E-mail: qili1020@tsinghua.edu.cn (Qi Li)

Supplementary Figures

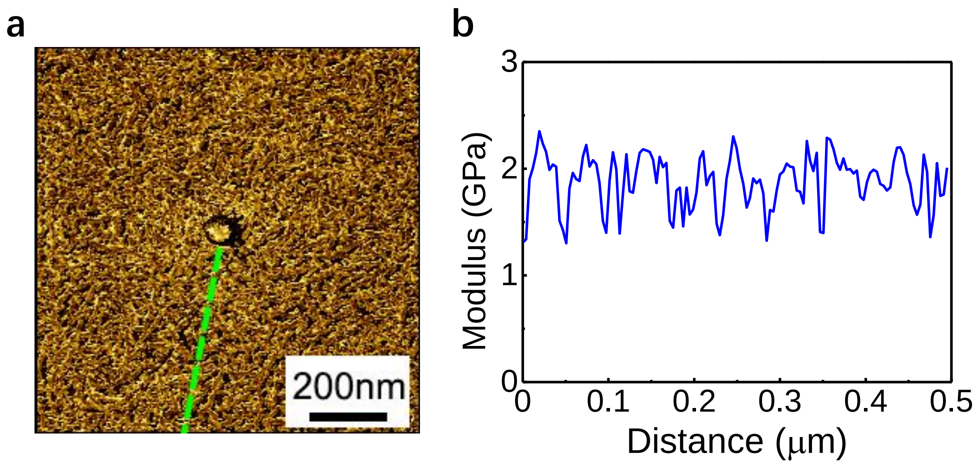

Fig. S1 a) The elastic modulus of sample with undecorated particles. **b)** Profile of **a)** at the dashed line

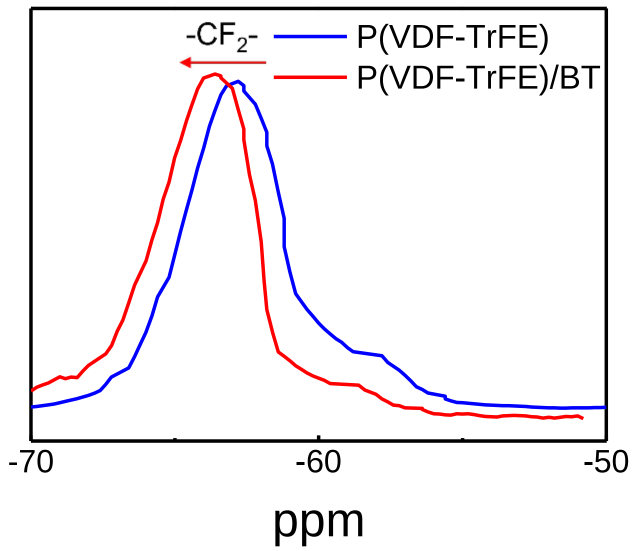

Fig. S2 ^{19}F NMR spectra of P(VDF-TrFE) and its nanocomposites

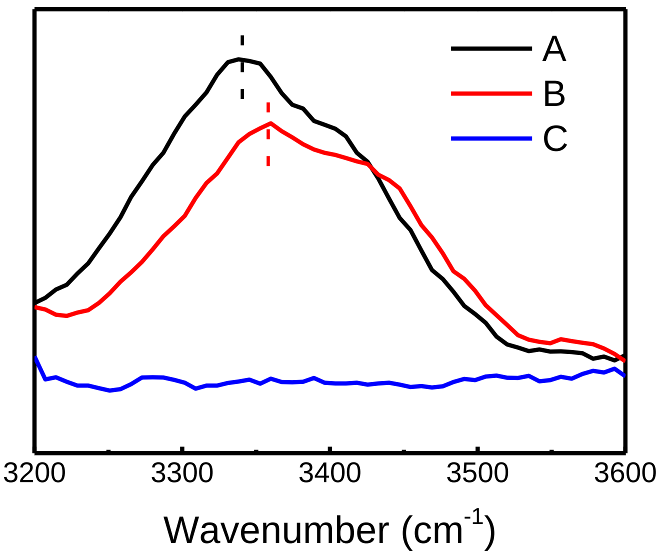

Fig. S3 Nano-IR spectra of different spots. A: the interface of BHEAPTS-decorated sample. B: the interface of TMPS-decorated sample. C: the matrix of BHEAPTS -decorated sample

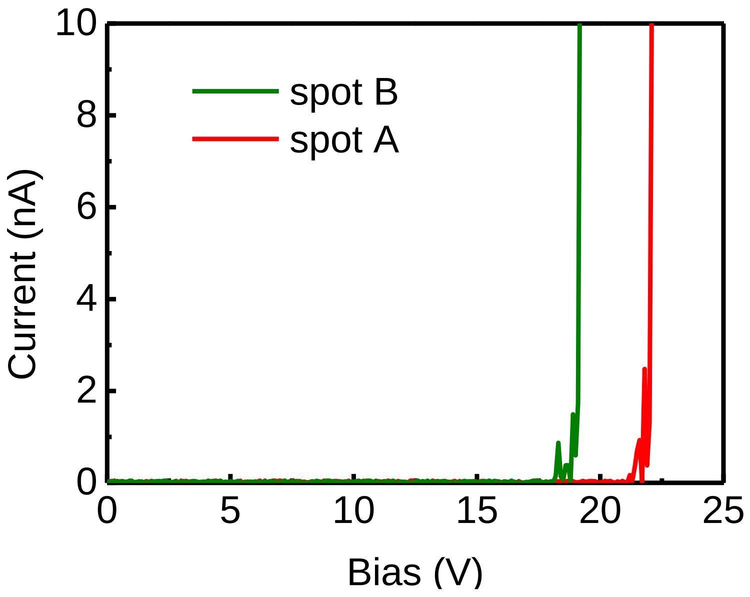

Fig. S4 Breakdown measurements by C-AFM of point A and point B in the BHEAPTS-decorated sample
